# Supplementary material for: Predictors of drug and substance abuse among school-going adolescents living in drug hotspot in Malaysia
Source: PLoS One. 2024 Jun 14;19(6):e0305460. doi: 10.1371/journal.pone.0305460 (PMC11178186; doi:10.1371/journal.pone.0305460)
Supplement: Attachment — Submitted filename: Response to Reviewers.docx [file pone.0305460.s001.docx]

REPLY TO THE REVIEWERS.

BASIC COMMENTS BY REVIEWERS

| BIL | COMMENT | REVIEWER 1 | REVIEWER 2 | NOTES |
| --- | --- | --- | --- | --- |
| 1 | Is the manuscript technically sound, and do the data support the conclusions?  -The manuscript must describe a technically sound piece of scientific research with data that supports the conclusions. Experiments must have been conducted rigorously, with appropriate controls, replication, and sample sizes. The conclusions must be drawn appropriately based on the data presented. | YES | YES | Thank you and we take note. |
| 2 | Has the statistical analysis been performed appropriately and rigorously? | YES | YES | Thank you. |
| 3 | Have the authors made all data underlying the findings in their manuscript fully available?  -The PLOS Data policy requires authors to make all data underlying the findings described in their manuscript fully available without restriction, with rare exception (please refer to the Data Availability Statement in the manuscript PDF file). The data should be provided as part of the manuscript or its supporting information, or deposited to a public repository. For example, in addition to summary statistics, the data points behind means, medians and variance measures should be available. If there are restrictions on publicly sharing data—e.g. participant privacy or use of data from a third party—those must be specified. | YES | YES | Thank you. |
| 4 | Is the manuscript presented in an intelligible fashion and written in standard English?  -PLOS ONE does not copyedit accepted manuscripts, so the language in submitted articles must be clear, correct, and unambiguous. Any typographical or grammatical errors should be corrected at revision, so please note any specific errors here. | YES | NO | Thank you for pointing this out. The article has been edited by a professional editor, however the manuscript has undergone the second revision by an international professional editor. |

DETAILS

CHECK LIST AFTER REVISION

| BIL | ITEM | NOTES |
| --- | --- | --- |
| 1 | A rebuttal letter that responds to each point raised by the academic editor and reviewer(s). You should upload this letter as a separate file labeled 'Response to Reviewers'. | A rebuttal letter has been prepared to respond to the reviewers. |
| 2 | A marked-up copy of your manuscript that highlights changes made to the original version. You should upload this as a separate file labeled 'Revised Manuscript with Track Changes'. | All changes has been highlighted in the revised manuscript |
| 3 | An unmarked version of your revised paper without tracked changes. You should upload this as a separate file labeled 'Manuscript'. | The unmarked version has been prepared. |

JOURNAL REQUIREMENT

| BIL | ITEM | NOTES |
| --- | --- | --- |
| 1 | When submitting your revision, we need you to address these additional requirements. Please ensure that your manuscript meets PLOS ONE's style requirements, including those for file naming. The PLOS ONE style templates can be found at <https://journals.plos.org/plosone/s/file?id=wjVg/PLOSOne_formatting_sample_main_body.pdf> and <https://journals.plos.org/plosone/s/file?id=ba62/PLOSOne_formatting_sample_title_authors_affiliations.pdf> | Thank you. The manuscript has been edited to meet the journal’s style requirements. |
| 2 | Thank you for stating the following financial disclosure: The authors acknowledge the Ministry of Higher Education Malaysia and The Universiti Kebangsaan Malaysia, (UKM) for funding this study under the Long-Term Research Grant Scheme-(LGRS/1/2019/UKM-UKM/2/1). We also thank the research team for their commitment and tireless efforts in producing the manuscript.  Please state what role the funders took in the study. If the funders had no role, please state: "The funders had no role in study design, data collection and analysis, decision to publish, or preparation of the manuscript." If this statement is not correct you must amend it as needed. Please include this amended Role of Funder statement in your cover letter; we will change the online submission form on your behalf | We noted this and have already edited it. |
| 3 | Thank you for stating the following in the Acknowledgments Section of your manuscript: The authors acknowledge the Ministry of Higher Education Malaysia and The Universiti Kebangsaan Malaysia, (UKM) for funding this study under the Long-Term Research Grant Scheme-(LGRS/1/2019/UKM-UKM/2/1). We also thank the research team for their commitment and tireless efforts in producing the manuscript.  We note that you have provided funding information that is not currently declared in your Funding Statement. However, funding information should not appear in the Acknowledgments section or other areas of your manuscript. We will only publish funding information present in the Funding Statement section of the online submission form. Please remove any funding-related text from the manuscript and let us know how you would like to update your Funding Statement.   Currently, your Funding Statement reads as follows: The authors acknowledge the Ministry of Higher Education Malaysia and The Universiti Kebangsaan Malaysia, (UKM) for funding this study under the Long-Term Research Grant Scheme-(LGRS/1/2019/UKM-UKM/2/1). We also thank the research team for their commitment and tireless efforts in producing the manuscript. Please include your amended statements within your cover letter; we will change the online submission form on your behalf | Thank you for your message regarding this matter. We have noted your guidance and have already removed the acknowledgement of funding form the main manuscript as per your instructions.  Our revised Funding Statement is as follows:  ‘The authors acknowledge the Ministry of Higher Education Malaysia and The Universiti Kebangsaan Malaysia, (UKM) for funding this study under the Long-Term Research Grant Scheme-(LGRS/1/2019/UKM-UKM/2/1). However, the funders had no role in study design, data collection and analysis, decision to publish, or preparation of the manuscript’ . |
| 4 | We note that your Data Availability Statement is currently as follows: All relevant data are within the manuscript and its Supporting Information files. Please confirm at this time whether or not your submission contains all raw data required to replicate the results of your study. Authors must share the “minimal data set” for their submission. PLOS defines the minimal data set to consist of the data required to replicate all study findings reported in the article, as well as related metadata and methods (https://journals.plos.org/plosone/s/data-availability#loc-minimal-data-set-definition). For example, authors should submit the following data:- The values behind the means, standard deviations and other measures reported;- The values used to build graphs;- The points extracted from images for analysis. Authors do not need to submit their entire data set if only a portion of the data was used in the reported study. If your submission does not contain these data, please either upload them as Supporting Information files or deposit them to a stable, public repository and provide us with the relevant URLs, DOIs, or accession numbers. For a list of recommended repositories, please see <https://journals.plos.org/plosone/s/recommended-repositories>  If there are ethical or legal restrictions on sharing a de-identified data set, please explain them in detail (e.g., data contain potentially sensitive information, data are owned by a third-party organization, etc.) and who has imposed them (e.g., an ethics committee). Please also provide contact information for a data access committee, ethics committee, or other institutional body to which data requests may be sent. If data are owned by a third party, please indicate how others may request data access. | The raw data set has been published in Mendeley Data. Here’s the URL for your reference : <https://data.mendeley.com/datasets/ggv2cd88my/2> |
| 5 | Please include captions for your Supporting Information files at the end of your manuscript, and update any in-text citations to match accordingly. Please see our Supporting Information guidelines for more information: <http://journals.plos.org/plosone/s/supporting-information>. | Done, thank you. |

REVIEW COMMENT TO AUTHOR

**REVIEWER 1**

**Comment 1:**

**Abtract**

This paper examines a convincing issue. Nevertheless, it has a few flaws. Abstracts must provide a concise introduction to the topic, describe how data were gathered, and identify the study site. Kindly explain the research methodology. It must condense the introduction to the issue while expanding the methodology description.

**Reply:** Thank you for your insightful comments on our manuscripts. We appreciate your acknowledgement of the issue we address in our paper and your constructive comments regarding its presentation. In response to your feedback, we have revised the abstract to ensure it meets the criteria you outlined, we have condensed the introduction to the issue while expanding the description of our research methodology. The adjustment as follow:

For the introduction to the topic, how data were gathered and the study site: The Malaysian Anti-drug Agency (AADK) has identified 155 hotspot areas across Malaysia of which 78 were randomly selected as the study sites. These areas were identified as having extreme drug-related activities such as drug trafficking and drug-related crimes. According to the literature, several factors influence adolescents to be involved in DSA. Therefore, understanding the risk factors in the context of Malaysian school-going adolescents is of utmost importance.

For the methodology, we added: Adolescents in the hotspot areas were selected by means of a cross-sectional survey design with a cluster sampling method. The sample comprised 3382 school-going adolescents, and the data were collected through face-to-face interviews. The logit model method with STATA software was used to analyse the data.

**Comment 2:**

**Introduction:**

Author is required to provide an explanation of the specific state or region in Malaysia that is classified as a drug-based hot spot. What initiatives has the relevant agency undertaken to mitigate the prevalence of drug-related hotspots in Malaysia? What type of incident, such as a drug-related criminal case, is occurring in the hotspot region? In the article, author describes older adolescents, male, and Western ethnicity are significant predictors of substance abuse on a global scale. How about the demography of Malaysia? Is it identical? From the present study in Malaysia, what others benefit in studying the factors contributing of drug abuse besides prioritizing of current intervention?

**Reply:**
Regarding the identification of drug-based hotspots in Malaysia, we have provided a clear explanation of the specific state or region classified as such. In text, we have added:

In Malaysia, a total of 155 drug-related hotspots have been identified by the AADK in 2020. This overall number of hotspots was found to have decreased from the total of 178 hotspots reported in 2018. All 155 of these areas encompass all localities in Peninsular Malaysia, including Sabah and Sarawak. The breakdown of the hotspots is as follows: Perlis (6 hotspots), Kedah (13), Pulau Pinang (7), Perak (14), Selangor (13), Federal Territory of Kuala Lumpur (8), Kelantan (15), Terengganu (15), Pahang (12), Melaka (10), Negeri Sembilan (6), Johor (17), Sarawak (7), Sabah (9) and Federal Territory of Labuan (3). These hotspots are focal points identified as grappling with drug addiction issues, with 60% of the entire nation’s population under surveillance, while also serving as regions contributing to social issues such as social tourism and drug-related crimes such as robbery and drug trafficking. The designation of a hotspot is based on four primary indicators: total of individuals under surveillance, total of suspected individuals, total of new addicts and community readiness level.

Additionally, we have elaborated on the initiatives undertaken by the relevant agencies to mitigate the prevalence of drug-related hotspots in Malaysia. In text, we have explained it as follows:

One of the strategies to minimize the drug-related activities by 2025 is ‘Hotspots Greening Program’ involving 155 hotspots in Malaysia, implemented collaboratively with government agencies and non-governmental organizations. The program’s strategies are a) preventing new drug addicts, b) maintaining recovery rates, c) increasing drug addict detection, d) enhancing cooperation and involvement of agencies or communities, and e) restricting drug entry.

**To answer to the question** ‘What type of incident, such as a drug-related criminal case, is occurring in the hotspot region’, we added:

These hotspots are focal points identified as grappling with drug addiction issues, with 60% of the entire nation’s population under surveillance, while also serving as regions contributing to social issues such as social tourism and drug-related crimes such as robbery and drug trafficking.

**For comment** ‘In the article, author describes older adolescents, male, and Western ethnicity are significant predictors of substance abuse on a global scale. How about the demography of Malaysia? Is it identical?’, we already confirmed the similarity with additional text as follows:

Globally, males, older adolescents, and Western ethnicity tend to report higher instances of drug use compared to other demographic groups. Similarly in Malaysia, older age groups (Ismail et al., 2022) and male adolescents have been found as more at risk (Hong & Peltzer, 2019; Institute for Public Health, 2017; Institute of Public Health, 2012) than the counterparts. In terms of race, over all Bumiputeras has been reported as the high-risk ethnic group in substance and drug use followed by Indians and Chinese compared to Malays although when it comes to individual substances different these ethnicities differ (Institute for Public Health, 2017; Rodzlan Hasani et al., 2021).

**For the comment** ‘From the present study in Malaysia, what others benefit in studying the factors contributing of drug abuse besides prioritizing of current intervention?’, we added the explanation as follows:

It is crucial to focus studies on hotspots to better understand their unique characteristics and the course of action needed to obtain effective preventive treatment. The efficacy of crime prevention of crime prevention strategies is augmented when directed towards identified hotspots and areas exhibiting heightened criminal activity (Weisburd et al., 2016). A crime prevention study focusing on 110 hotspots identified led to a significant reduction in crime within eight months (Koper, 2005).

And,

The research will elucidate several potential predictors comprising socio-economic factors, peer influences, family dynamics, educational experiences, access to drugs and community characteristics. By considering this broad spectrum of variables, we aim to construct a comprehensive and holistic picture of the factors that contribute to drug abuse among Malaysian school-going adolescents in hot-spot areas. Through in-depth understanding of these predictors, we can develop targeted and context-specific strategies to prevent and address substance abuse in these high-risk communities (Substance Abuse and Mental Health Services Administration US, 2016).

The significance of this research lies in its potential to address several critical issues. Firstly, it enables a nuanced understanding of the challenges faced by adolescents in drug-based hot-spot areas, recognizing that their experiences may differ subtly or significantly from those in other regions. Secondly, it provides a foundation for evidence-based policymaking and intervention design, ensuring that resources are allocated efficiently and effectively to combat substance abuse in the areas where it is most prevalent and crucial. Ultimately, the findings from this study hold the promise of making a substantial contribution to the ongoing efforts to address substance abuse in Malaysia. Understanding the complex interplay of factors in these high-risk communities, we aspire to empower policymakers, educators, and healthcare professionals with the knowledge needed to develop targeted interventions, reduce substance abuse rates, and promote the well-being and prospects of adolescents in these vulnerable areas.

**Comment 3:**

Does this research involve face to face interviews? Is that the extent of the researcher's training?

**Reply:** Thank you for your inquiry regarding our research methodology. Yes, our research involves face-to-face interviews as part of the data collection process. In our manuscript, we already added the explanation as follows:

Face-to-face interviews and questionnaires were administered by the trained researcher. Training has been provided intensively by project leader to research assistants to ensure a good understanding of interview protocols, and interview simulations have also been conducted.

**Comment 4:**

The study also reveals that adolescents often exhibit externalizing behaviors such as aggression and rule-breaking, which are strongly associated with an elevated risk of engaging in drug-use (Fite at al., 2021: Mathias et al., 2015 & Niv et al., 2013). This statement refers to the male or female in the context of demography?

**Reply:** In the context of our study, the statement concerning adolescents exhibiting externalizing behaviors and their association with an elevated risk of engaging in drug use primarily refers to the male population.

**Comment 5:**
Rational conclusions are reached. Nevertheless, the challenges associated with cultivating constructive peer networks, equipping parents with information and assistance, carrying out impactful public health campaigns, executing focused interventions, and establishing systems for assessing programmes appear to be widely discussed in the current literature. Which additional policies must be considered within the Malaysian context?

**Reply:** In response to your inquiry, we believed that within the Malaysian context, additional policies should be considered to address the multifaceted nature of substance abuse prevention and intervention. We added:

These findings can be utilized to strengthen the existing policy measures for adolescents to initiate a drug free community. These main implications of the study include strengthening awareness, early identification, and intervention for adolescents and parents through universal school-based programs and targeted programs. A universal program such as social emotional learning programs can be introduced into school curriculum and extracurricular activities from early adolescence onwards which involves learning emotion management, healthy coping skills, and interspersal skills. Drugs and substance harm awareness programs can be aimed for middle adolescents and parents. Additionally, strengthening the current ongoing random screening system that is only limited to urine tests can be supplemented with adding a layer to school-based screening for identifying students with emotion management difficulty and interpersonal skill deficit. Furthermore, implementing targeted programs such as skills groups and individual sessions for at risk adolescents through school counsellors and a referral process along with targeted interventions for adolescents involved in substance with the help of out-patient experts can be implemented. Additionally, engaging adolescents in school to enhance academic and vocational training can be beneficial. These interventions will help in building individual psychological capital, to equip adolescents with skills to deal with social environmental influences, and early identification of at-risk individual to provide support with and a layer of support for individuals involving in substance use.

**Comment 6:**

In conclusion, evaluate the strengths and weaknesses of this study.

**Reply:** In the manuscript, we added:

The current study has certain strengths. The main strength of the study is that it is a nationwide study based on hot spots. It also provides insightful understanding regarding the drug using adolescents in the high-risk areas.

And,

The strengths of the study include nationwide data and discovery of useful knowledge on the current situation. Nevertheless, the data were self-reported and focusing on school-going adolescent may potentially leave out the high-risk adolescents who have dropped out of schools.

**REVIEWER 2**

**Comment 1**: The introduction already explained the background of study. However, there are only 10 empirical references been referred with repeating the same 3 authors; Brook, Ismail, Nawi. Thus, the author need to add more empirical references.

**Reply:** We add some new references in the introduction part. Kindly refer the citation as follows:
Hong & Peltzer, 2019; Institute for Public Health, 2017; Institute of Public Health, 2012, Rodzlan Hasani et al., 2021, Weisburd et al., 2016, Koper, 2005

**Comment 2:**

This is a good study, but the article is insufficient to explain the background of study in introduction part. Please explain the theoretical background of the study.

**Reply:** Thank you for your compliment. We already have added our theoretical backgrounds of the study. You may find it in our manuscript as follows:

The investigation into the predictors of current DSA among school-going adolescents in the drug hotspots of Malaysia was underpinned by a synthesis of the pertinent theoretical frameworks in social sciences and public health. Grounded in the social learning theory (Bandura, 1977), the study endeavoured to elucidate the mechanisms through which adolescents acquire attitudes and behaviours towards DSA by observing and imitating their social group (Miller & Carroll, 2006; Misra & Cooper, 2013; Shanmugam, 2017), encompassing peers (Simons-Morton & Chen, 2006; Reed et al., 2007) and familial influences (Alhammad et al., 2022).

Concurrently, the risk and protective factors framework served as an analytical scaffold to delineate the multifaceted interplay of individual, familial, peer, educational, and community-level determinants impinging upon the vulnerability or resilience of adolescents vis-à-vis DSA. Augmenting this inquiry, the ecological systems theory (Bronfenbrenner, 1994) was invoked to explore the nested ecological contexts encompassing microsystemic, mesosystemic, exosystemic, and macrosystemic influences, which collectively mould the drug-related behaviours of adolescents.

**Comment 3:**

The instruments like ASSIST version 3.0 and YSR need to explain more than current.

**Reply**: We already have explained it in the current version of the manuscript. Kindly refer to the ‘study instrument’ section.

**Comment 4:**

It is confusing to interpret the results of the sample characteristics and their involvement in drug abuse. The first paragraph shows that the majority of respondents had experience with drug use, but this finding was not consistent with the other reported data. This part needs to be re-examined.

**Reply:** We appreciate your scrutiny of this aspect of our study. In the revised version of our manuscript, we have addressed this inconsistency and provided clarification to ensure coherence and accuracy in the reporting of results.

**Comment 5:**

The report in drug users by ethnic is hard to understand.

**Reply:** In response to your comment, we have revised the report on drug users by ethnicity to provide a clearer and more concise presentation of our findings. Thank you.
